# Protein Kinase D1 (PKD1) Phosphorylation Promotes Dopaminergic Neuronal Survival during 6-OHDA-Induced Oxidative Stress

**DOI:** 10.1371/journal.pone.0096947

**Published:** 2014-05-07

**Authors:** Arunkumar Asaithambi, Muhammet Ay, Huajun Jin, Anamitra Gosh, Vellareddy Anantharam, Arthi Kanthasamy, Anumantha G. Kanthasamy

**Affiliations:** Department of Biomedical Sciences, Iowa State University, Ames, Iowa, United States of America; Emory University, United States of America

## Abstract

Oxidative stress is a major pathophysiological mediator of degenerative processes in many neurodegenerative diseases including Parkinson’s disease (PD). Aberrant cell signaling governed by protein phosphorylation has been linked to oxidative damage of dopaminergic neurons in PD. Although several studies have associated activation of certain protein kinases with apoptotic cell death in PD, very little is known about protein kinase regulation of cell survival and protection against oxidative damage and degeneration in dopaminergic neurons. Here, we characterized the PKD1-mediated protective pathway against oxidative damage in cell culture models of PD. Dopaminergic neurotoxicant 6-hydroxy dopamine (6-OHDA) was used to induce oxidative stress in the N27 dopaminergic cell model and in primary mesencephalic neurons. Our results indicated that 6-OHDA induced the PKD1 activation loop (PKD1S744/S748) phosphorylation during early stages of oxidative stress and that PKD1 activation preceded cell death. We also found that 6-OHDA rapidly increased phosphorylation of the C-terminal S916 in PKD1, which is required for PKD1 activation loop (PKD1S744/748) phosphorylation. Interestingly, negative modulation of PKD1 activation by RNAi knockdown or by the pharmacological inhibition of PKD1 by kbNB-14270 augmented 6-OHDA-induced apoptosis, while positive modulation of PKD1 by the overexpression of full length PKD1 (PKD1^WT^) or constitutively active PKD1 (PKD1^S744E/S748E^) attenuated 6-OHDA-induced apoptosis, suggesting an anti-apoptotic role for PKD1 during oxidative neuronal injury. Collectively, our results demonstrate that PKD1 signaling plays a cell survival role during early stages of oxidative stress in dopaminergic neurons and therefore, positive modulation of the PKD1-mediated signal transduction pathway can provide a novel neuroprotective strategy against PD.

## Introduction

Parkinson’s disease (PD) is a major neurodegenerative disorder affecting over a million Americans at an annual cost of several billion dollars. The incidence of PD is projected to increase dramatically with the advancing median age of the U.S. population. This creates a scientific imperative to improve our understanding of the causes and medical management of PD. Several studies have shown that oxidative stress is one of the major factors underlying the etiology of age-related neurodegeneration. Experimental findings from cell cultures, animal models, and humans indicate that oxidative stress and apoptosis may contribute to the pathophysiological processes in PD [1], [2]. Apoptosis, resulting from altered cellular redox, is a continual cell death process involving multiple signaling molecules, and therefore, identification of the key molecules contributing to the apoptotic cell death process in dopaminergic neurons might provide novel therapeutic targets [1], [3]–[5]. To study key signaling molecules involved in oxidative stress-induced neuronal apoptosis in PD, we used the dopaminergic system specific neurotoxicant 6-hydroxydopamine (2,4,5-trihydroxyphenylethylamine; 6-OHDA). 6-OHDA has been shown to recapitulate key pathophysiological changes of PD, including mitochondrial dysfunction and oxidative stress [6], [7]. When 6-OHDA is oxidized in dopaminergic neurons, it produces reactive oxygen species resulting in redox imbalance, which activate various signaling molecules in dopaminergic neurons. However, key intrinsic signaling molecules, which contribute to increased vulnerability of dopaminergic neurons to oxidative damage, are not well understood. We have previously shown that oxidative stress mediated by 6-OHDA can induce caspase-3-mediated proteolytic cleavage of PKCδ, which then mediates apoptotic cell death in cell culture and animal models of PD [8].

Protein kinase D (PKD) belongs to the calcium/calmodulin-dependent protein kinase (CAMK) family and consists of three isoforms that are homologous in structure and function, namely, PKD1, PKD2 and PKD3. Among PKDs, PKD1 is the most studied member of the family, and is widely expressed in many tissues and organs, including thyroid, brain, heart and lungs [9], [10]. The regulatory domain of PKD1 is comprised of two cysteine-rich domains that bind diacylglycerol, and a pleckstrin homology (PH) domain that has an autoinhibitory function [10]. PKD1 can be activated by phosphorylation at the dual serine residues (ser 744/748 in mouse) in the activation loop by the members of the PKC family, depending upon the cellular type and stimuli [11]–[13]. The other protein kinases, including novel PKCs, such as PKC (δ, ε, η, and θ), have been shown to regulate PKD1 activation [13]. The activation loop phosphorylation leads to subsequent autophosphorylation at other sites, such as ser 916, which has been used as a measure of PKD1 activation [10], [12]. Signaling through PKD1 is activated in response to multiple stimuli, and its activation has been shown to play important roles in diverse cellular functions, including proliferation, cytoskeletal reorganization, Golgi function, immune function, and cell survival [14]–[21]. The biological functions of PKD1 in the nervous system are not well studied. In the present study, we demonstrate that the 6-OHDA-activated PKD1 signaling pathway serves as a key compensatory protective mechanism in dopaminergic neurons during the early stages of oxidative insult and dopaminergic degeneration in cell culture models of PD.

## Materials and Methods

### Cell Culture

The immortalized dopaminergic neuronal cell line obtained from rat mesencephalon, (1RB_3_AN_27_; referred to as N27 cells), which was described previously [22], [23], was a kind gift from Dr. Kedar N. Prasad (University of Colorado Health Sciences Center, Denver, CO). N27 cells were cultured in RPMI 1640 medium containing 10% fetal bovine serum, 2 mM l-glutamine, 50 units of penicillin, and 50 µg/ml streptomycin. Cells were kept in a humidified atmosphere of 5% CO_2_ at 37°C, as described previously [22]. Various research groups use N27 cells as a cell culture model for PD [24]–[30].

### Primary Mesencephalic Neuronal Culture

All of the procedures involving animal handling were approved by the Institutional Animal Care Use Committee (IACUC) at the Iowa State University. The ventral mesencephalon of gestational 16- to 18-day-old mouse embryos was used to prepare primary mesencephalic neuronal cultures [31], [32]. Tissues obtained from E16 to E18 mouse embryos were dissociated in Hanks’ balanced salt solution containing trypsin-0.25% EDTA for 30 min at 37°C. The dissociated cells were then plated at equal density (0.5×10^6^ cells) on 0.1 mg/ml poly-D-lysine precoated 12 mm coverslips. Neurobasal medium fortified with B-27 supplements, 500 µM l-glutamine, 100 IU/ml penicillin, and 100 µg/ml streptomycin (Invitrogen) was used to grow the cells. The cells were maintained in a humidified CO_2_ incubator (5% CO_2_ and 37°C). Approximately 5- to 6-day-old cultures were used for experiments. Primary mesencephalic neuronal cells were exposed to 10 µM 6-OHDA for 1–3 h.

### Treatment Paradigm

N27 cells were exposed to 6-OHDA (100 µM) for 0–9 h at 37°C. Primary neurons were exposed to 6-OHDA (10 µM) for 1–3 h. We determined that 100 µM 6-OHDA is an optimal dose in N27 cells based on our preliminary dose-response studies. Also, we and many other laboratories [8], [33]–[37] showed that 6-OHDA selectively targets dopaminergic neurons at concentrations of 10–100 µM because it enters dopaminergic neurons through dopamine transporter. In this regard, dopamine transporters are expressed at much higher levels in primary mesencephalic dopaminergic neurons relative to N27 cells. Thus, primary dopaminergic neurons were treated with a lower dose of 6-OHDA (10 µM). Cell lysates were used for Western blotting and immunoprecipitation studies. Untreated cells were grown in complete medium and used as the experimental control.

### Cytotoxicity Assays

Cytotoxicity of N27 cells against 6-OHDA (100 µM) was determined using the Sytox green cytotoxicity assay, as described previously [8], [38]. This cytotoxicity assay was optimized for a multiwell high-throughput format, which is more efficient and sensitive than other cytotoxicity measurements [8], [38]. Briefly, N27 cells were grown in 24-well cell culture plates at 100,000 cells per well and treated with 6-OHDA (100 µM) along with 1 µM Sytox green fluorescent dye. The Sytox green assay allows dead cells to be viewed directly under a fluorescence microscope. Furthermore, the fluorescence can be quantitatively measured using a fluorescence microplate reader (excitation 485 nm; emission 538 nm) (Biotek). Phase contrast and fluorescent images were taken after 6-OHDA exposure using a NIKON TE2000 microscope coupled with a SPOT digital camera.

### Immunocytochemistry

After 6-OHDA treatment, primary mesencephalic neurons were fixed using 4% paraformaldehyde. Non-specific sites were blocked with 2% bovine serum albumin, 0.5% Triton and 0.05% Tween-20 in phosphate-buffered saline (PBS). The cells then were incubated with anti-TH, anti-native PKD1 and anti-PKD1-pS744/S748 primary antibodies in PBS containing 1% BSA at 4°C overnight. This was followed by incubation with Alexa 488 and Alexa 568 conjugated secondary antibodies in PBS containing 1% BSA. Further incubation was done with nuclear Hoechst 33342 dye to stain the nucleus. After further washing, the coverslips were mounted on slides, and viewed under an inverted fluorescence microscope (Nikon TE-2000U, Tokyo, Japan). The images were captured with a SPOT digital camera (Diagnostic Instruments, Inc., Sterling Heights, MI).

### Western Blot Analysis

Cells were lysed in either modified RIPA buffer or M-PER buffer (Thermo Scientific) for Western blot, immunoprecipitation and kinase assays. Nuclear and cytoplasmic extracts were isolated using the NE-PER nuclear and cytoplasmic extraction kit (Thermo Scientific), as described previously [39]. PKD1 polyclonal (1∶1000), PKD1-pS744/S748 (1∶1000), PKD1-pS916 (1∶1000), Lamin B (1∶1000), α-Tubulin (1∶5000) and β-actin (1∶10000) antibodies were used to blot the membranes. IR dye-800 conjugated anti-rabbit (1∶5000) and Alexa Fluor 680 conjugated anti-mouse (1∶10000) were used for antibody detection with the Odyssey IR Imaging system (LICOR), as previously described [40], [41].

### Transient Transfections

HA-tagged full-length human PKD1 WT and mutant plasmids were obtained from Addgene, Inc. [42]. Electroporation was carried out with an Amaxa Nucleofector transfection instrument, as per the manufacturer’s protocol. The transfected cells were then transferred to T-75 flasks or 6-well plates, as desired, and allowed to grow for a 24 h period before the treatment.

### RNAi

Predesigned PKD1-siRNA was purchased from IDT, Inc. PKD1-siRNA effectively suppressed >80% of PKD1 protein expression levels after a 36 h post-transfection. N27 cells (50–70% confluence) were transfected with siRNA duplexes using an Amaxa Nucleofector kit (Amaxa), as described in our previous study [39], [43].

### Statistical Analysis

Data analysis was done using Prism 3.0 software (GraphPad Software, San Diego, CA). Bonferroni’s multiple comparison procedure was applied to test for significant differences between treatment and control groups. Differences with p<0.05, p<0.01, and p<0.001 were considered significant from two or more independent experiments (n≥6), and are indicated in the figures.

## Results

### 6-OHDA Induces Cytotoxic Cell Death of N27 Dopaminergic Cells

**Figure 1:**
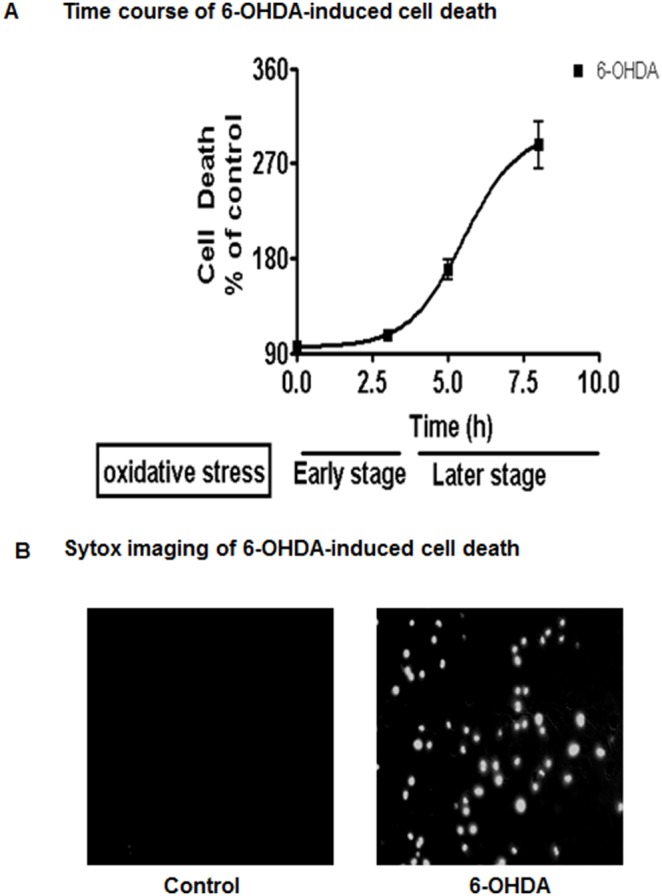
6-OHDA-induced oxidative stress causes cell death in N27 dopaminergic neuronal cell models. N27 dopaminergic cells were treated with 6-OHDA (100 µM) for 0–8 h and assayed for cytotoxicity using Sytox green fluorescence assay. The fluorescence was measured by using a fluorescence plate reader. Non-linear regression was performed from two or more independent experiments (A), and visualized by fluorescence microscopy (B).

We first determined the time course of N27 dopaminergic neuronal cells to 6-OHDA-induced neurotoxicity using the Sytox cell death assay. The N27 cells were treated with 100 µM 6-OHDA and the cell death was monitored up to 8 h. As shown in Fig. 1A, 6-OHDA induced cytotoxic cell death in a time-dependent manner beginning at 3 h. 6-OHDA induced more than a 3-fold increase in cell death at 8 hr. Visualization of Sytox-stained N27 cells with fluorescence microscopy further confirmed the sensitivity of cells to 6-OHDA-induced cytotoxicity (Fig. 1B). These results suggest that 6-OHDA induces cytotoxic cell death in dopaminergic neuronal cells in a time-dependent manner.

### 6-OHDA Induces Activation Loop Phosphorylation of PKD1 in N27 Dopaminergic Cells

**Figure 2:**
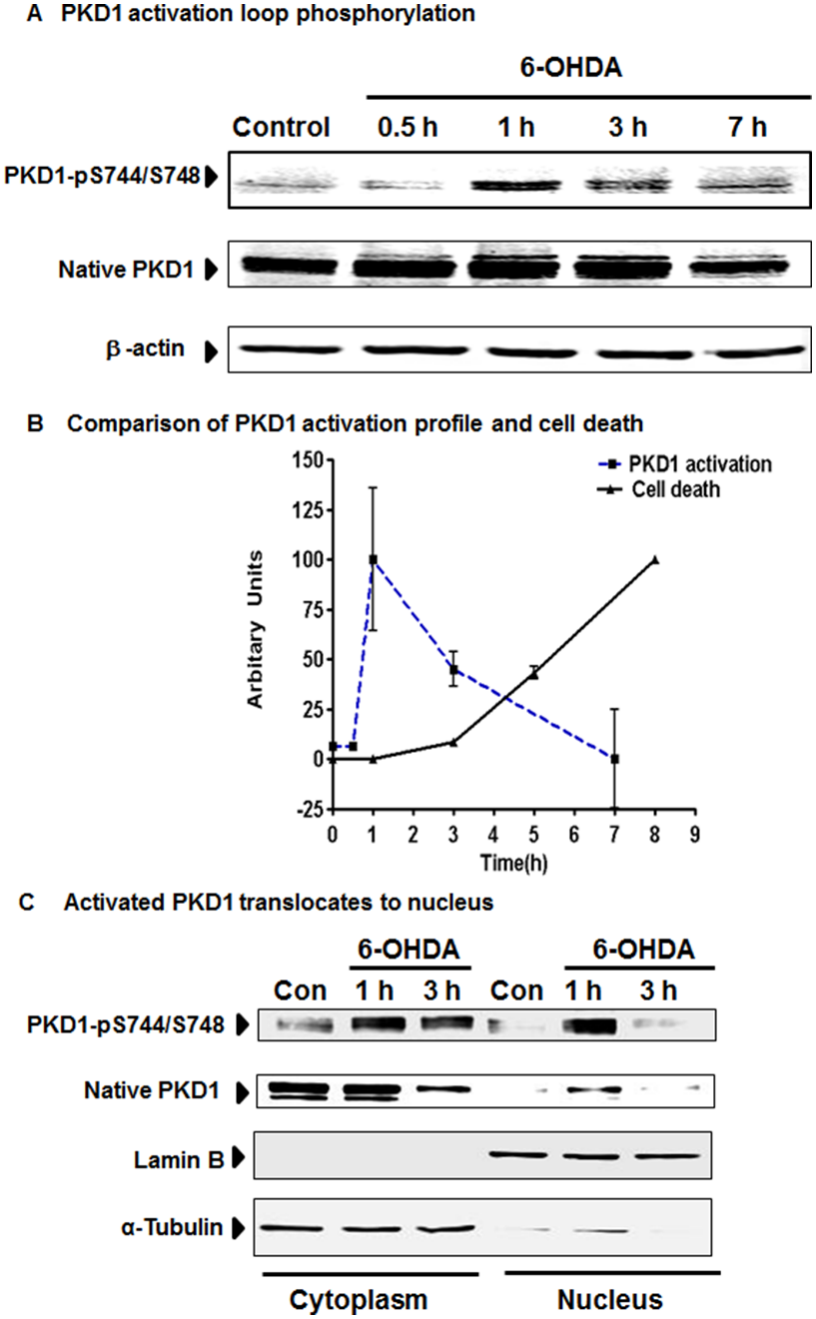
PKD1 is activated in N27 cells during 6-OHDA-induced oxidative stress. N27 dopaminergic neuronal cells were treated with 6-OHDA (100 µM) for 1–7 h and probed for PKD1 activation loop phosphorylation pS744/pS748 and native PKD1 expression (A). A comparative time course graph depicting the reciprocal relationship between PKD1 activation and cytotoxicity during 6-OHDA exposure (B). Cytoplasmic and nuclear extracts from control and 6-OHDA-treated N27 cells were prepared and probed for PKD1 activation loop phosphorylation pS744/pS748 and native PKD1 expression (C). Lamin B (nuclear fraction) or α-Tubulin (cytoplasmic fraction) was used as loading control.

Next, we measured PKD1 phosphorylation of Ser744/Ser748 and compared it with cytotoxic cell death. Phosphorylation of Ser744/Ser748 located in the activation loop of PKD1 results in its activation. Therefore, we determined if 6-OHDA induces activation loop phosphorylation of PKD1 in N27 dopaminergic cells. We used antibody directed against phosphorylated Ser744/Ser748 in PKD1 to determine PKD1 activation. As shown in Fig. 2A, PKD1 activation occurs in a time-dependent manner from 0–7 h. The phosphorylated Ser744/Ser748 PKD1, 110 kDa band, peaked at 1 h and returned to basal levels over 7 h. Densitometry analysis revealed that 6-OHDA increased the PKD1 Ser744/Ser748 phosphorylation dramatically within 1 h, then started declining at 3 h and finally returned to control levels around 7 h (Fig. 2B). Thus, the 6-OHDA-induced phosphorylation of PKD1 Ser744/Ser748 occurred at early stages of oxidative insult and preceded cytotoxic cell death. As shown in Fig. 2B, comparison of the time course of the 6-OHDA-induced cytotoxicity with PKD1 Ser744/Ser748 phosphorylation showed an inverse relationship, suggesting that PKD1 activation may serve as a compensatory protective response against oxidative insult.

We also performed Western blot analysis following nuclear/cytoplasmic fractionation to determine the subcellular distribution of PKD1 phosphorylation. As depicted in Fig. 2C, in untreated N27 cells, native PKD1 and phosphorylated Ser744/Ser748 PKD1 were primarily located in the cytoplasm, which is consistent with the general concept that PKD1 is a predominantly cytoplasmic protein [10]. 6-OHDA treatment rapidly increased PKD1 Ser744/Ser748 phosphorylation in the cytosol. Interestingly, phosphorylated Ser744/Ser748 PKD1 also readily translocated to the nucleus, indicating that oxidative insult promotes a rapid PKD1 nuclear translocation in dopaminergic cells. Consistent with the data in Fig. 2B, this nuclear translocation of phosphorylated Ser744/Ser748 PKD1 peaked at 1 h after 6-OHDA treatment, whereas PKD1 phosphorylation in both cytoplasm and nucleus reduced 3 h post-treatment.

### Ser 916 Phosphorylation Precedes Activation Loop Phosphorylation of PKD1 in N27 Dopaminergic Cells

**Figure 3:**
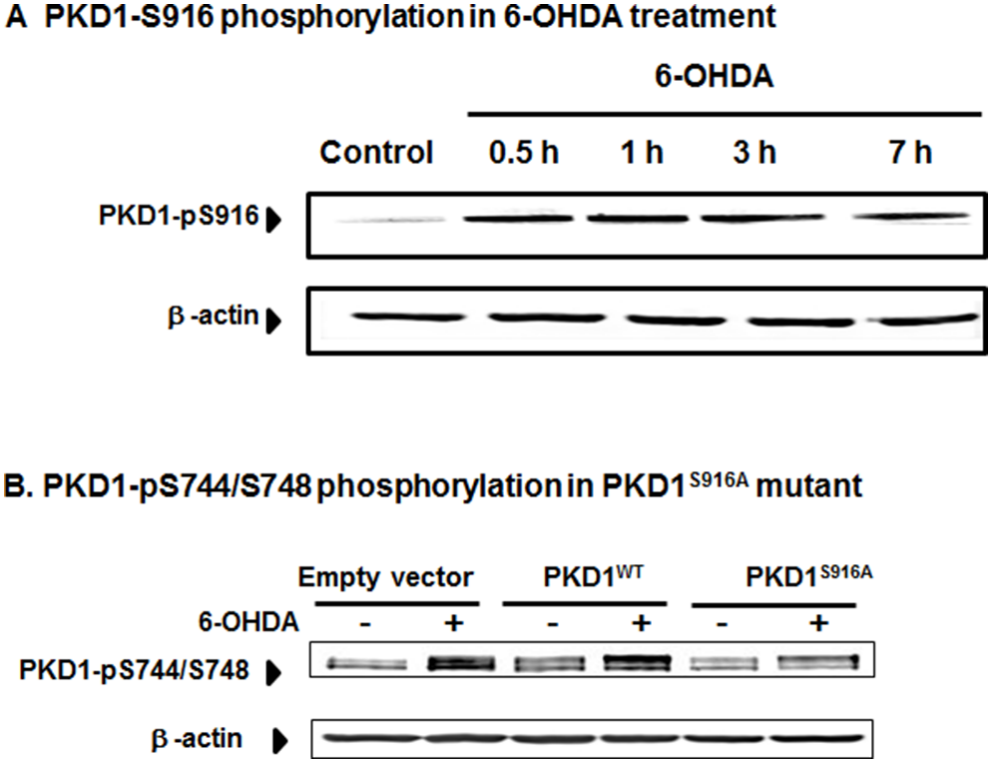
PKCδ-dependent S916-phosphorylation of PKD1 precedes PKD1 S744/S748 active loop phosphorylation in N27 dopaminergic cells. N27 dopaminergic cells were treated with 100 µM 6-OHDA for 1–7 h and monitored for PKD1 S916 phosphorylation (A). N27 cells transiently expressing PKD1^S916A^ mutant prevent PKD1 activation during oxidative stress, as seen by Western blotting for PKD1 S744/S748 phosphorylation (B).

Following the characterization of activation loop PKD1 (pS744/pS748) phosphorylation, we further examined whether additional phosphorylation occurs during 6-OHDA-induced oxidative stress. Since PKD1 Ser 916 phosphorylation site in the C-terminal region serves as an important regulator of PKD1 activation, we investigated the role of PKD1 Ser 916 phosphorylation during 6-OHDA treatment. As shown in Fig. 3A, exposure to 100 µM 6-OHDA induced PKD1 Ser 916 phosphorylation as early as 30 min, and preceded activation loop PKD1 (pS744/pS748) phosphorylation (Fig. 2A). Next, we examined whether PKD1 916 phosphorylation regulates the PKD1 (pS744/pS748) phosphorylation during oxidative stress. For this study, we overexpressed the phosphorylation defective mutant PKD1^S916A^ and the native wild type PKD1^WT^ in N27 dopaminergic cells by transient transfection and then exposed the cells to 100 µM 6-OHDA for 1 h. As shown in Fig. 3B, again, we observed 6-OHDA-induced PKD1 (pS744/pS748) activation loop phosphorylation in empty vector-transfected cells. However, overexpression of PKD1^S916A^ but not PKD1^WT^ blocked this effect, suggesting that Ser 916 phosphorylation is required for PKD1 activation loop phosphorylation. Together, these results suggest that C-terminal PKD1 Ser916 phosphorylation occurs at very early stages of oxidative stress to positively regulate PKD1 (pS744/pS748) activation loop phosphorylation during 6-OHDA-induced oxidative stress in dopaminergic cells.

### Pharmacological Inhibition and RNAi Mediated Suppression of PKD1 Exacerbates 6-OHDA-Induced Cell Death in N27 Dopaminergic Cells

**Figure 4:**
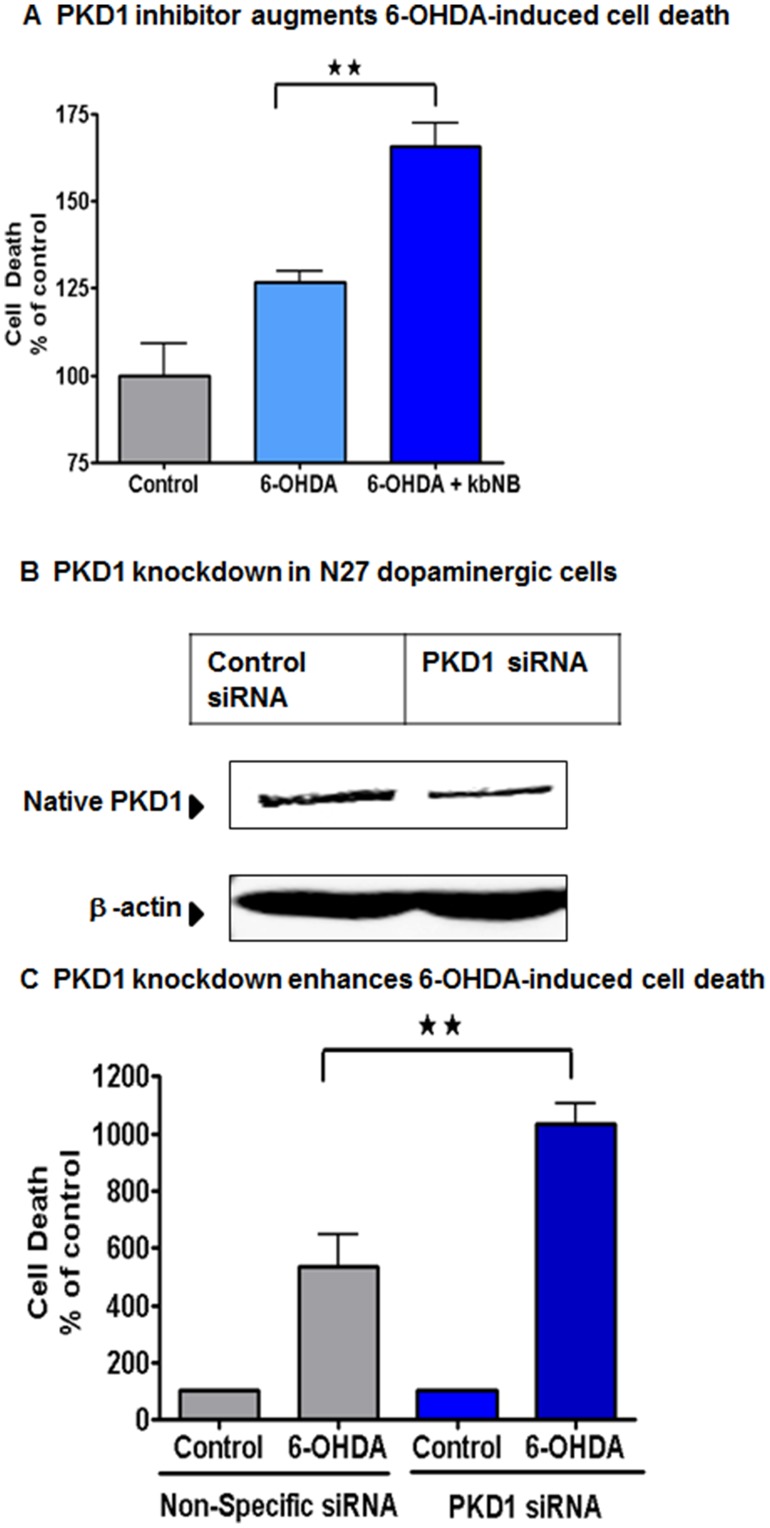
PKD1 activation is essential for cell survival during 6-OHDA-induced oxidative stress in N27 dopaminergic neurons. N27 cells were co-treated with the PKD1 inhibitor kb-NB 14270 and monitored for cell death during 6-OHDA treatment for 4 h (A). N27 dopaminergic cells were transfected with 1 µM PKD1 siRNA and non-specific siRNA (B) and treated with 100 µM 6-OHDA for 9 h and monitored for cytotoxicity using Sytox green assay. Fluorescence measurements for the incorporation of Sytox green dye were performed using a fluorescence plate reader. **, p<0.01 denotes a significant difference between non-specific siRNA-6-OHDA and PKD1 siRNA-6-OHDA treated groups (C).

In order to determine the functional role of PKD1 activation during oxidative stress-induced dopaminergic cell death, we first used a pharmacological approach that involved the allosteric PKD inhibitor kbNB 142–70. Briefly, N27 dopaminergic cells were exposed to 100 µM 6-OHDA in the presence or absence of 50 µM kbNB 142–70. We then determined cytotoxic cell death using the Sytox green assay. As shown in Fig. 4A, co-treatment with 50 µM kbNB 142–70 exacerbated 6-OHDA-induced cytotoxic cell death by more than 30%, as compared to 6-OHDA-alone treated cells. To further confirm the cell survival role of PKD1, we used siRNA directed against the coding region of PKD1 to suppress its expression and then monitored cell death using the Sytox cell death assay. N27 dopaminergic cells were transfected with PKD1 siRNA and non-specific siRNA, and then 24 h post-transfection, cells were exposed to 100 µM 6-OHDA. PKD1 expression was significantly suppressed in PKD1 siRNA transfected compared to non-specific siRNA-transfected N27 dopaminergic cells (Fig. 4B). As revealed by the cell death assay (Fig. 4C), 6-OHDA-induced neurotoxic cell death increased 10-fold in PKD1 siRNA transfected cells compared to 5-fold in non-specific siRNA transfected dopaminergic cells at 9 hr. Together, these results demonstrate that the negative modulation of PKD1 using pharmacological and siRNA approaches results in exacerbated oxidative stress-induced cell death, suggesting a cell survival role for PKD1.

### Overexpression of Wild-type and Constitutively Active PKD1 Protects against 6-OHDA-Induced Cytotoxic Cell Death in N27 Dopaminergic Cells

**Figure 5:**
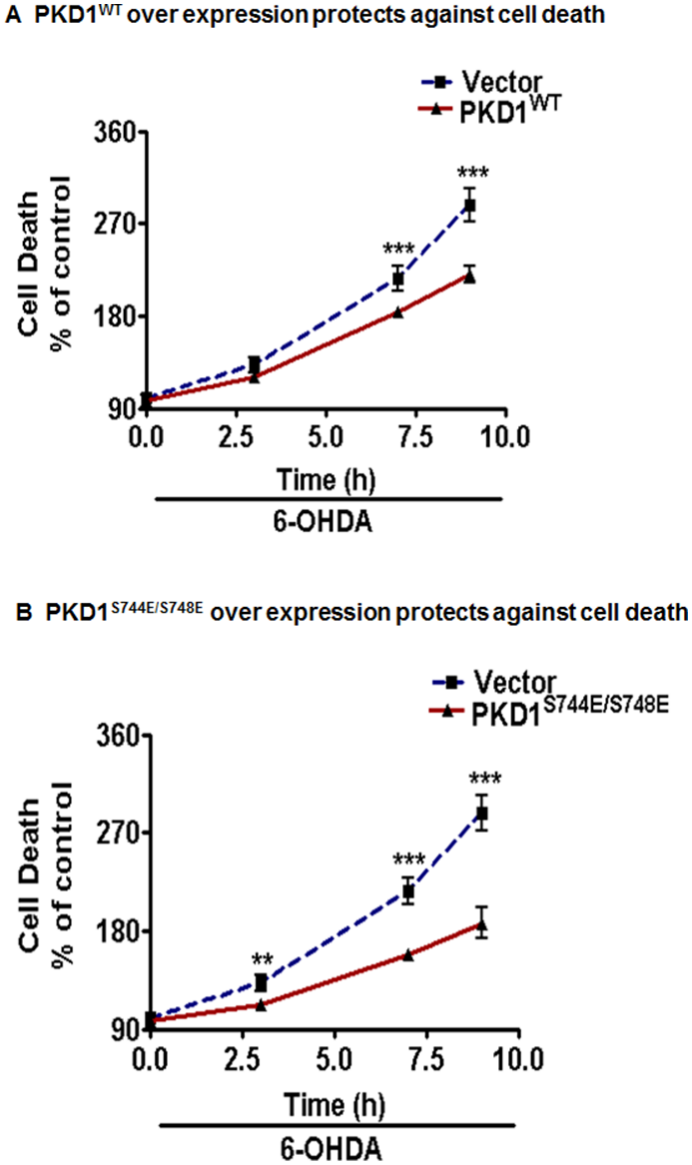
Modulation of PKD1 offers neuroprotection in N27 dopaminergic neuronal cells. N27 dopaminergic cells transiently transfected with 5 µg of full-length PKD1 plasmid (PKD1^WT^) and 5 µg of vector plasmid were treated with or without 100 µM 6-OHDA and monitored for cytotoxicity at various time points using Sytox green assay. ***, p<0.001 denotes a significant difference between treatment groups from n≥6 (A). N27 dopaminergic cells transiently transfected with 5 µg of constitutively active PKD1^S744E/S748E^ mutant and 5 µg of vector plasmid were treated with or without 100 µM 6-OHDA and monitored for cytotoxicity at various time points using Sytox green assay. ***, p<0.001 denotes a significant difference between treatment groups from n≥6 (B).

To unequivocally confirm the cell survival role of PKD1, we overexpressed the full- length wild-type PKD1 plasmid (PKD1^WT^) and the constitutively active PKD1^S744E/S748E^ in N27 dopaminergic cells, which were then exposed to 6-OHDA. Mutation of serine residues at positions 744 and 748 results in constitutively active PKD1 [44]. As shown in Fig. 5A, overexpression of PKD1^WT^ significantly attenuated 6-OHDA-induced cytotoxic cell death in a time-dependent manner compared to vector-transfected dopaminergic cells. Similarly, overexpression of constitutively active PKD1^S744E/S748E^ also significantly attenuated 6-OHDA-induced cytotoxic cell death in a time-dependent manner compared to vector transfected dopaminergic cells (Fig. 5B). These results further validated a cell survival role as well as a protective compensatory role for PKD1 signaling during oxidative damage in dopaminergic neuronal cells.

**Figure 6:**
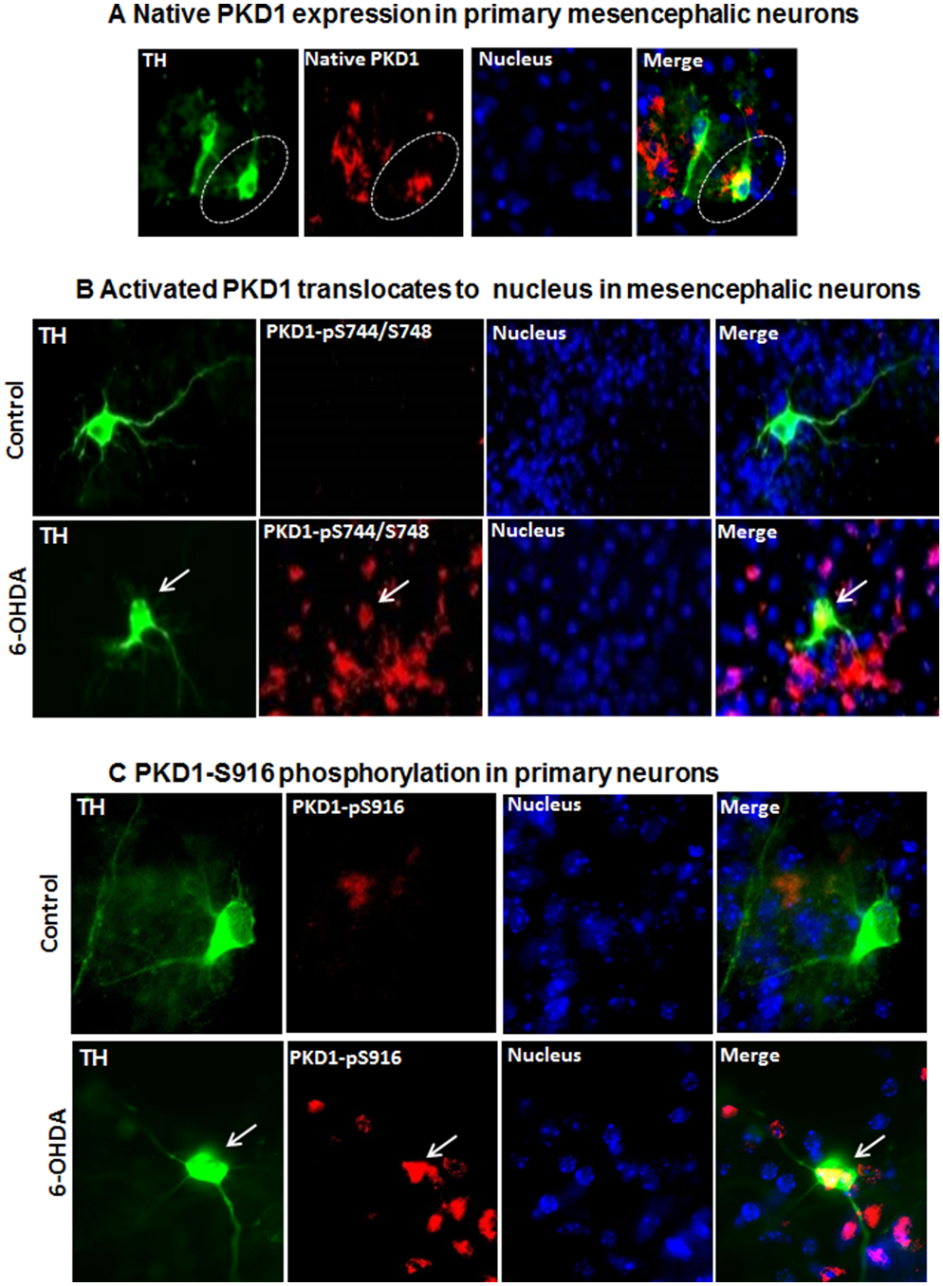
PKD1 is highly expressed in primary dopaminergic neurons and activated during 6-OHDA-induced oxidative stress. (**A**) Primary dopaminergic neurons stained for tyrosine hydroxylase (TH) show co-localization of native PKD1 with TH in the cytosol. TH-Green, PKD1 native-Red, Nucleus-Blue, Merge-Yellow. Nuclei were stained with Hoechst dye. (B) Immunofluorescence analysis of primary dopaminergic neurons stained for TH shows translocation of activated PKD1 to the nucleus during 6-OHDA exposure. TH-Green, PKD1pS744/S748-Red, Nucleus-Blue, Merge-Pink. Nuclei were stained with Hoechst dye. White arrows indicate the TH^+^ neurons, in which activated PKD1 translocates into nucleus after 6-OHDA treatment. (C) Primary dopaminergic neurons staining for TH show the presence of PKD1pS916 in both the cytosol and nucleus during 6-OHDA exposure. TH-Green, PKD1pS916-Red, red/yellow-Merge.

### 6-OHDA Induces Activation Loop Phosphorylation of PKD1 in Primary Mesencephalic Neurons

Finally, we examined if PKD1 activation occurs in primary mesencephalic dopaminergic neurons exposed to 6-OHDA. Immunocytochemical staining was performed to examine the subcellular localization of PKD1 in primary mesencephalic neurons. We found a high-level expression of PKD1 (red, Fig. 6A) in the cytosolic region of tyrosine hydroxylase-positive (TH^+^) primary dopaminergic neurons obtained from mouse mesencephalon (green, Fig. 6A). We also observed PKD1 expression in some of the non-dopaminergic neurons. Next, we performed immunocytochemical staining to examine the localization pattern of activated PKD1 following 6-OHDA treatment. Immunocytochemical staining for activated PKD1 showed that the activated PKD1 (pS744/pS748) (red) co-localized with the nuclear Hoechst stain (blue) during 10 µM 6-OHDA-induced oxidative stress in primary mesencephalic neurons, as visualized by fluorescence microscopy (Fig. 6B). Next, the effect of 6-OHDA on PKD1 Ser916 phosphorylation was also examined in primary mesencephalic neurons. We treated mouse primary mesencephalic cultures with 6-OHDA (10 µM), and then PKD1 Ser916 phosphorylation immunoreactivity of TH-positive neurons was determined. As shown in Fig. 6C, immunocytochemical staining showed that PKD1pS916 (red) was localized (pink/yellow) in both the cytosol and nuclei of TH^+^ (green) primary mesencephalic neurons during 10 µM 6-OHDA treatment. Together, these results indicate that activated PKD1 translocates to the nucleus of primary dopaminergic neurons when treated with a Parkinsonian specific toxicant like 6-OHDA, and that 6-OHDA also induces PKD1 S916 phosphorylation in primary dopaminergic neurons.

## Discussion

This study reveals the existence of a novel defensive signaling mechanism in cell culture models of PD during neurotoxic stress. Our study reports, for the first time, three key findings in an oxidative stress-induced dopaminergic degeneration model using 6-OHDA: (i) PKD1 activation occurs at an early stage of oxidative damage in cell culture models of PD, (ii) phosphorylation of Ser 916 residue regulates activation loop phosphorylation (PKD1- pS744/S748), and (iii) modulation of PKD1 signaling has protective effects against oxidative damage of dopaminergic cell death.

Oxidative stress triggers apoptosis and causes neurodegeneration through activation of multiple signaling molecules including kinases and proteases [5], [45]–[47]. MAP kinases, including JNK and p38 kinase as well as their downstream effectors, have been well studied in apoptotic neuronal cell death [48], [49], however, signaling mechanisms governing the compensatory protective response during early stages of neurotoxic insult are yet to be defined. In particular, key protein kinases signaling involved in the protective function are still being investigated [50], [51]. PKD1 has been identified as an important survival signaling transduction kinase associated with oxidative stress in non-neuronal cell lines [20], [52]–[54]. It has been shown that oxidative stress induces PKD1 activation via activation loop phosphorylation pS744/pS748, and other PKC isoforms, including PKCδ, have been shown to mediate PKD1 activation loop phosphorylation in non-neuronal systems [54]–[58]. The role of PKD1 in the brain, especially in dopaminergic degeneration, remains unknown. Specifically, the relationship between PKD1 signaling and neuronal survival has not yet been examined in detail.

**Figure 7:**
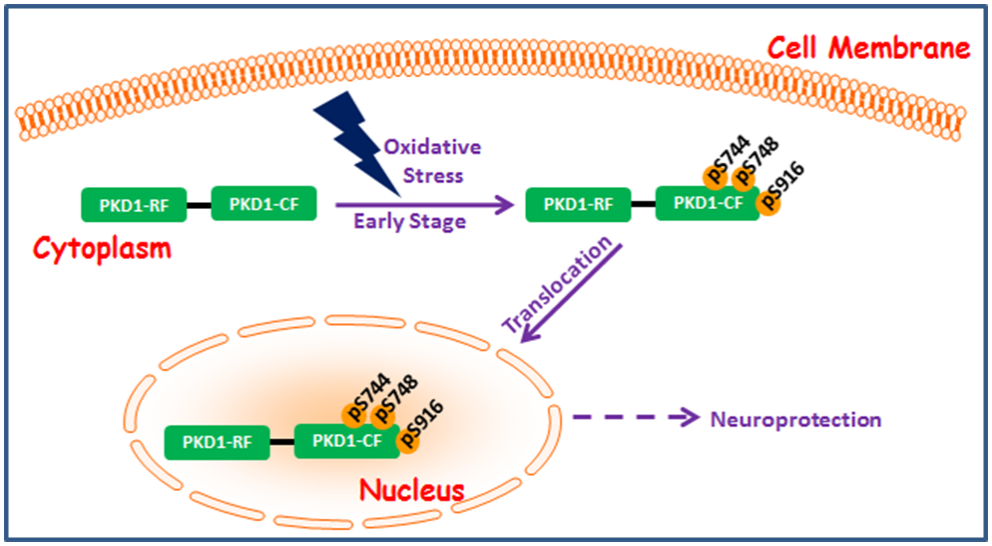
A proposed model for PKD1 neuroprotection in dopaminergic neurons during early stages of oxidative insult. Oxidative insult rapidly induces PKD1 activation loop phosphorylation at pS744/pS748, which then translocates from the cytoplasm to the nucleus. The PKD1 activation and nuclear translocation lead to counteracting oxidative injury and subsequent initiation of cell survival processes.

In the current study, we explored the relationship between PKD1 signaling and dopaminergic degeneration in cell culture models of PD. Several studies have demonstrated DNA fragmentation during late stages of 6-OHDA-induced apoptotic cell death. DNA fragmentation is preceded by ROS generation, mitochondrial dysfunction, and caspase-3 activation [34], [59], [60]. We have recently shown ROS generation, mitochondrial dysfunction and caspase-3-mediated proteolytic activation of PKCδ play a critical role in 6-OHDA-induced dopaminergic degeneration in cell culture and animal models of PD [8]. In the present study, we report the activation of PKD1 during the early stages of 6-OHDA-induced oxidative stress in cell culture models of PD. Multiple phosphorylation sites have been implicated in PKD1 activation loop phosphorylation, depending on the cell types and stimuli [20], [61]. Previous groups have shown the involvement of Ser 916 phosphorylation in PKD1 activation in non-neuronal models [57], [62], [63]. We demonstrate that S916 phosphorylation is a prerequisite for PKD1ser744/Ser748 activation loop phosphorylation [64]. Our data suggest phosphorylation of C-terminally located Ser 916 results in unmasking of the Ser744/Ser748 residues located in the activation loop for subsequent phosphorylation and PKD1 activation during 6-OHDA-induced cytotoxicity. Furthermore, both pharmacological and genetic inhibition exacerbated 6-OHDA-induced dopaminergic cell death, suggesting that PKD1 is a cell survival kinase. Comparison of PKD1 activation and 6-OHDA-induced cytotoxicity shows that PKD1 activation peaks during the early oxidative stress stage when no measurable cytotoxicity occurs. Interestingly, when PKD1 activation begins to decline at the end of the early stage oxidative stress, cell death begins to occur. When the constitutively active PKD1 mutant (PKD1^S744E/S748E^) or wild-type PKD1 plasmid (PKD1^WT^) is overexpressed, dopaminergic cells are resistant to 6-OHDA-induced cytotoxicity, even during the late stages of oxidative stress, which is consistent with our hypothesis that PKD1 activation protects against oxidative damage in dopaminergic cells. Furthermore, we also observed 6-OHDA treatment induced nuclear translocation of phosphorylated PKD1 to possibly regulate the transcription of cell survival transcription factors and genes in dopaminergic cell death. Our results are consistent with recent studies demonstrating nuclear translocation of PKD1 in B cells, cardiomyocytes and oestoblasts [20], [65]–[67]. Furthermore, we believe that the protective effect of PKD1 signaling should not be limited to dopaminergic neurons because PKD1 is also highly expressed in other cell types, including cardiomyocytes and prostate and testis germ cells [10]. Therefore, any cells expressing a sufficient level of PKD1 can adopt Ser-744/748 and Ser-916 phosphorylation-dependent activation mechanisms to protect against oxidative stress. A proposed model for PKD1’s neuroprotection based on our experimental results is illustrated in Figure 7. Our future studies will explore the PKD1 mediated nuclear events that might regulate the early compensatory neuroprotective effects during oxidative stress in dopaminergic neurons.

In conclusion, our results demonstrate the existence of a novel protective signaling mechanism mediated by PKD1 in dopaminergic neurons. During the early stage of 6-OHDA-induced oxidative stress, PKD1 is activated and protects neurons against oxidative damage. However, prolonged oxidative insult inactivates PKD1 signaling, which might lead to neuronal death. Thus, positive modulation promotes neuronal survival while negative modulation of PKD1 augments cell death. Overall, our findings suggest that PKD1 may function as a cell survival switch in dopaminergic neurons, and its modulation could be a novel neuroprotective strategy against oxidative damage in PD.
